# Hormonal variation and temporal dynamics of musth in Asian elephants (*Elephas maximus*) are associated with age, body condition and the social environment

**DOI:** 10.1093/conphys/coad019

**Published:** 2023-04-25

**Authors:** Chase A LaDue, Kathleen E Hunt, Wendy K Kiso, Elizabeth W Freeman

**Affiliations:** Department of Environmental Science and Policy, 4400 University Drive, MSN 5F2, George Mason University, Fairfax, VA 22030, USA; Oklahoma City Zoo and Botanical Garden, 2000 Remington Place, Oklahoma City, OK 73111, USA; Department of Biology, George Mason University, 10900 University Boulevard, Manassas, VA 20110, USA; Smithsonian-Mason School of Conservation, 1500 Remount Road, Front Royal, VA 22630, USA; White Oak Conservation Foundation, 581705 White Oak Road, Yulee, FL 32097, USA; Colossal Biosciences, 3309 Elm Street, Dallas, TX 75226, USA; School of Integrative Studies, 4400 University Drive, MSN 5D3, George Mason University, Fairfax, VA 22030, USA

**Keywords:** welfare, thyroid hormones, sustainability, musth, health, glucocorticoids, androgens

## Abstract

The sustainability of endangered Asian elephants in human care is threatened in part by low breeding success and concerns over individual animal wellbeing. Male elephants have received less research attention compared to females, yet males deserve special consideration due to their unique reproductive biology (particularly the sexual state of “musth”) and the complex interaction of physiological, environmental, and social pressures they face. We measured fecal androgen metabolites (FAMs), fecal glucocorticoid metabolites (FGMs), and fecal triiodothyronine metabolites (FT3s) collected weekly over approximately 12 months from 26 male Asian elephants housed in zoos across the US, hypothesizing that FAM, FGM, and FT3 concentrations would be associated with temporal correlates of musth and would vary further with intrinsic (musth status, age, body condition) and extrinsic (social environment) factors. The duration of each musth episode was positively associated with exposure to male conspecifics and negatively associated with body condition. Further, elevated FAM concentrations were associated with social exposure, age, and body condition, and FGM concentrations also varied with age and body condition. FT3 concentrations were not associated with any factor we measured. We also identified periods of lower FAM concentration than confirmed musth episodes (but still higher than baseline FAM concentrations) that we termed “elevated FAM episodes.” The durations of these episodes were negatively correlated with exposure to other male elephants. Together, these results provide evidence that hormone profiles (including those that are predicted to change around musth) vary significantly between male Asian elephants in a way that may be attributed to intrinsic and extrinsic factors. Studies like these serve to enhance the sustainability of *ex-situ* populations by providing wildlife managers with information to enhance the health, welfare, and reproduction of threatened species like Asian elephants.

## Teaser

Asian elephants are endangered, and males require special consideration to enhance the sustainability and well-being of zoo populations. Measurements of androgen, glucocorticoid and thyroid hormone metabolites in 26 male Asian elephants revealed that metabolite concentrations varied with sexual state, age, body condition and exposure to other elephants.

## 1. Introduction

Zoological facilities are critically important to the continued existence of threatened species (Conde *et al.,* 2011), including Asian elephants (*Elephas maximus*) (Riddle and Stremme, 2011; Bechert *et al.,* 2019). Asian elephants are endangered (Williams *et al.,* 2020), and out of the approximately sixty thousand remaining Asian elephants in the world (Menon and Tiwari, 2019), it is estimated that as many as 30% of these exist in human care, including in zoos, private ownership, circuses, camps, and other similar wildlife parks and operations (Riddle and Stremme, 2011). Unfortunately, the sustainability of many *ex-situ* elephant populations is at risk (Wiese, 2000; Thitaram, 2012), with limited reproductive success due to factors including complicated breeding logistics and a small number of suitable candidates (Schmitt, 2006; Kiso *et al.,* 2007; Brown *et al.,* 2016), and fatal hemorrhagic disease associated with elephant endotheliotropic herpesvirus infection (Long *et al.,* 2015; Zachariah *et al.,* 2018). There also exists concern over the wellbeing of individual elephants due to the species’ cognitive and sensory complexities, extensive social capacity, and charismatic nature (de Silva and Wittemyer, 2012; Albert *et al.,* 2018; Jacobson and Plotnik, 2020; Schulte and LaDue, 2021; Stoeger, 2021; van Water *et al.,* 2022). As such, animal-centered strategies that target both welfare and long-term sustainability challenges for *ex-situ* Asian elephant populations are warranted.

Historically, captive elephant populations have been female-biased, as female elephants were preferentially imported from range countries due to their tractability and often without the intent to establish *ex-situ* captive breeding programs (Rees, 2009). However, there is now decreased reliance on wild importations, with populations relying almost entirely on *ex-situ* breeding efforts (Wiese and Willis, 2006; Clubb *et al.,* 2009; Scherer *et al.,* 2022). However, with enhanced breeding success and survivorship in *ex-situ* populations, the proportion of male elephants is increasing markedly. For instance, while there is still a strong female bias in the North American Asian elephant population [26% of the population (*n* = 41) is male], approximately 50% of Asian elephants born in North America since the species first arrived on the continent (*n* ≈ 226 elephants) have been males (unpublished data, Association of Zoos and Aquariums’ Elephant Taxon Advisory Group, 2022). This poses significant husbandry and management challenges not previously encountered by most facilities, especially as much of the research on elephant sustainability has focused on issues and complexities related to reproduction in females (Freeman *et al.,* 2009; Dow *et al.,* 2011; Morfeld and Brown, 2014; Brown *et al.,* 2016), and facilities may be limited by infrastructure that has centered around female-specific needs. Clearly, there are opportunities to better understand topics related to male elephant management that will have implications for the short- and long-term sustainability of *ex-situ* Asian elephant populations.

In the long-term, we should continue to engage more male elephants in breeding to reproduce naturally and/or to act as semen donors for artificial insemination, as many calves produced in *ex-situ* facilities have been sired by relatively few males (Nordin, 2017; Schmitt, 2022). While the historical lack of breeding opportunities may partially explain this pattern, poor semen quality is also common in zoo-housed males (Kiso *et al.,* 2007; Kiso *et al.,* 2011). Androgens (e.g. testosterone) help to regulate spermatogenesis and sperm development (Smith and Walker, 2014), and mature male Asian elephants undergo a unique sexually active state called “musth” that is characterized by elevated androgen concentrations (Jainudeen *et al.,* 1972a; Rasmussen *et al.,* 1984; Lincoln and Ratnasooriya, 1996). Musth is accompanied by a range of behavioral and physiological changes (Chave *et al.,* 2019; LaDue *et al.,* 2022a; LaDue *et al.,* 2022c) that can last from a few days to several months in Asian elephants (Scott and Riddle, 2003; LaDue *et al.,* 2014). Prolonged elevations in androgen concentrations that help to define musth begin to occur around the time of sexual maturity (Sukumar, 2003; Brown, 2014); but shorter, sporadic androgen spikes may occur in males around puberty and/or in response to social and/or sexual stimulation (Cooper *et al.,* 1990; Rasmussen *et al.,* 2002). While musth is not essential for successful reproduction to occur [i.e. non-musth males produce viable sperm (Kiso *et al.,* 2007)], it does facilitate inter- and intrasexual selection (LaDue *et al.,* 2022b) and is commonly observed in zoo-housed elephants (Scott and Riddle, 2003; LaDue *et al.,* 2014). Historically, elephants in musth have not been directly managed for reproductive procedures (e.g. for semen collection), and musth males may behave unpredictably around conspecifics when introduced for breeding (Scott and Riddle, 2003; Olson, 2004; LaDue *et al.,* 2014). Further, there may exist other intrinsic factors (e.g. age, health, metabolism) and extrinsic factors (e.g. physical environment, social environment) that influence androgen patterns, as has been described in other wild mammals (Liptrap and Raeside, 1968; Liptrap and Raeside, 1978; Borg *et al.,* 1991; Sands and Creel, 2004; Mooring *et al.,* 2006) and potentially elephants (Brown *et al.,* 2007; Chave *et al.,* 2019; Campbell *et al.,* 2022). Thus, identification of such factors that influence androgen secretion and/or musth in male Asian elephants would have strong implications for the sustainability of *ex-situ* populations.

Two other hormone classes also influence the short- and long-term wellbeing of male Asian elephants and may also be of relevance for understanding musth. The hypothalamic–pituitary–adrenal (HPA) axis in part regulates the stress response in vertebrates, releasing glucocorticoids (e.g. cortisol, corticosterone) from the adrenal glands after exposure to a variety of stressors to enact a range of short- and long-term responses (Sapolsky *et al.,* 2000). Further, thyroid hormones [e.g. triiodothyronine (T3) and thyroxine (T4)] facilitate transitions between metabolic and/or sexually active states in many mammals (Ryg and Langvatn, 1982; Eales, 1988; Loudon *et al.,* 1989; Shi and Barrell, 1994). Concentrations of both glucocorticoids and thyroid hormones may be expected to change around musth, as musth is thought to be both physiologically stressful and metabolically limited (Jainudeen *et al.,* 1972b; Dickerman *et al.,* 1994). Indeed, these hormones can vary with musth status in male elephants housed in zoos (Brown *et al.,* 2007; Chave *et al.,* 2019; Glaeser *et al.,* 2022; LaDue *et al.,* 2022a). However, glucocorticoids and thyroid hormones can also be affected by other factors, including the physical environment (Kumar *et al.,* 2014; Boyle *et al.,* 2015; Norkaew *et al.,* 2019; Bansiddhi *et al.,* 2019b; Szott *et al.,* 2020a; Szott *et al.,* 2020b), social dynamics (Schmid *et al.,* 2001; Burks *et al.,* 2004; Pokharel *et al.,* 2019a; Glaeser *et al.,* 2020; Glaeser *et al.,* 2022), and nutritional shifts that affect health and/or body condition (Pokharel *et al.,* 2017; Norkaew *et al.,* 2019; Pokharel *et al.,* 2019b).

The purpose of this study was to characterize hormonal variation in relation to intrinsic (age, body condition, musth status) and extrinsic factors (social environment) in zoo-housed Asian elephants via longitudinal analysis of fecal hormone metabolites: fecal androgen metabolites (FAM; e.g. testosterone and related fecal metabolites), fecal glucocorticoid metabolites (FGM; e.g. corticosterone, cortisol, and related fecal metabolites), and fecal T3 metabolites (FT3). Non-invasive sampling via feces allowed us to include animals from which it is logistically challenging to collect regular blood samples, and fecal hormone metabolites have been successfully measured in male Asian elephants before (Ghosal *et al.,* 2013; Pokharel *et al.,* 2017; Vijayakrishnan *et al.,* 2018; Brown *et al.,* 2019; Norkaew *et al.,* 2019; Bansiddhi *et al.,* 2019a; de Andrés *et al.,* 2021; Seltmann *et al.,* 2022). We hypothesized that the physiological and temporal correlates of musth would vary between the male elephants in our study. Further, we predicted that FAM, FGM, and FT3 concentrations would vary with age, body condition, and social environment, and that these factors also would be associated with the temporal variation of musth episodes. Hormone data from the individuals in this study have been presented in LaDue *et al.* (2022a), in which we correlate hormonal changes to physical and behavioral indicators of musth in wild and zoo-housed elephants. The present study investigates previously published data in the new context of how a male’s condition and the social environment within zoos may further influence hormonal activity in male Asian elephants.

## 2. Materials and Methods

### 2.1. Study sites and subjects

We sampled 26 male Asian elephants at ten facilities throughout the US, aged 9.2 to 57.0 years (mean ± SE age = 26.7 ± 3.1 years), from July 2019 to March 2021 (Table 1). Six of the elephants were born in the wild [ages of these elephants were estimated to the nearest year (Nordin, 2017)], and the rest were captive-born with known birthdates. Over the sample collection period, we asked each facility to note any major changes that occurred (e.g. births, deaths, and transfers of conspecifics; changes to exhibit spaces; major medical procedures). Husbandry conditions—including diet, feeding schedules, training, and enrichment—were consistent within each elephant over the study. Sample collection protocols were approved by George Mason University’s IACUC (1168839-1), the Association of Zoos and Aquariums’ Elephant Taxon Advisory Group and by the research committees at each participating facility.

**Table 1:** Details on each elephant included in this study. Animals marked with † were born in the wild, and so birthdates were estimated to the nearest year; all other animals were born in captivity. The number of adult males (including self), adult females and juveniles and calves residing at each facility during the study period are indicated. Sampling schedule is indicated by the month of first sample, month of last sample and the total number of samples collected per elephant. The number of confirmed musth episodes and elevated FAM episodes are also provided (see text for definitions).

Facility	Animal	Birthdate (D-M-Y)	Adult males	Adult females	Juv. + calves	First sample (M-Y)	Last sample (M-Y)	Samples collected	Confirmed musth episodes	Elevated FAM episodes
A	A1	19-Jan-09	9	23	2	Oct-19	Mar-21	50	1	4
A	A2^†^	1-Jan-71	9	23	2	Oct-19	Mar-21	42	5	0
A	A3^†^	1-Jan-73	9	23	2	Oct-19	Mar-21	52	1	1
A	A4	18-Nov-01	9	23	2	Oct-19	Mar-21	55	0	0
A	A5	1-Jun-05	9	23	2	Oct-19	Mar-21	53	1	1
A	A6	16-Aug-99	9	23	2	Oct-19	Mar-21	50	2	0
A	A7	21-May-02	9	23	2	Oct-19	Feb-21	48	0	0
A	A8^†^	1-Jan-63	9	23	2	Oct-19	Mar-21	52	1	1
B	B1	26-Jan-88	1	3	0	May-19	Jul-20	47	3	0
C	C1	27-Mar-09	2	4	0	Jul-19	Aug-20	46	0	0
C	C2	16-Jan-88	2	4	0	Jul-19	Aug-20	47	1	0
D	D1	17-Feb-08	5	0	0	Jul-19	Jul-20	56	1	2
D	D2	16-Apr-04	5	0	0	Jul-19	Jul-20	56	2	0
D	D3	15-Jul-08	5	0	0	Jul-19	Jul-20	57	0	0
D	D4^†^	1-Jan-71	5	0	0	Jul-19	Jul-20	57	1	3
D	D5	2-Nov-09	5	0	0	Jul-19	Jul-20	56	0	0
E	E1	4-Apr-91	2	3	2	Jun-19	Aug-20	60	1	1
E	E2	10-Jan-93	2	3	2	Jul-19	Aug-20	58	2	1
F	F1	4-May-10	3	4	3	Jun-19	Apr-20	31	0	0
F	F2^†^	1-Jan-65	3	4	3	Jul-19	Apr-20	32	0	0
F	F3	12-May-05	3	4	3	May-19	Jun-20	35	0	1
G	G1	2-Jul-81	1	5	0	Aug-19	Aug-20	49	0	0
H	H1	25-Nov-01	2	3	2	Jul-19	Aug-20	50	3	0
H	H2^†^	1-Jan-68	2	3	2	Jul-19	Aug-20	50	3	0
I	I1	8-May-97	1	5	2	Jun-19	Aug-20	61	2	3
J	J1	27-Dec-92	1	7	2	Jul-19	Sep-20	59	2	1

### 2.2. Sample collection

Zoo staff were asked to collect a fecal sample one time per week from each of the study elephants for twelve months, beginning around July 2019. However, logistical challenges resulting from the COVID-19 pandemic altered the ability of some facilities to regularly collect samples beginning around March 2020, and as a result several of these facilities extended the collection period to obtain more samples on otherwise underrepresented elephants. The average ± SE number of samples collected per elephant was 50.3 ± 1.6 (min = 31, max = 61), for a time span of 14.0 ± 0.4 mos (min = 9.3 mos, max = 16.7 mos). This resulted in 1309 total fecal samples included in this study.

For each sample collection, zoo staff collected ≈500 g of fecal material from the middle of a bolus, thereby minimizing contamination that may occur on the outside of a bolus. Staff were asked to collect fecal material from multiple places within the bolus, as metabolites are not equally distributed upon defecation (Brown *et al.,* 1994; Millspaugh and Washburn, 2004). All samples were collected within two hours of observed defecation, stored in labeled plastic bags and frozen at—20°C immediately after collection. Within three months of collection, samples were shipped overnight on ice to George Mason University (GMU, Fairfax, VA) for analysis. Upon receipt, all samples were stored at—20°C until extraction, which occurred within 12 months of collection.

**Figure 1:**
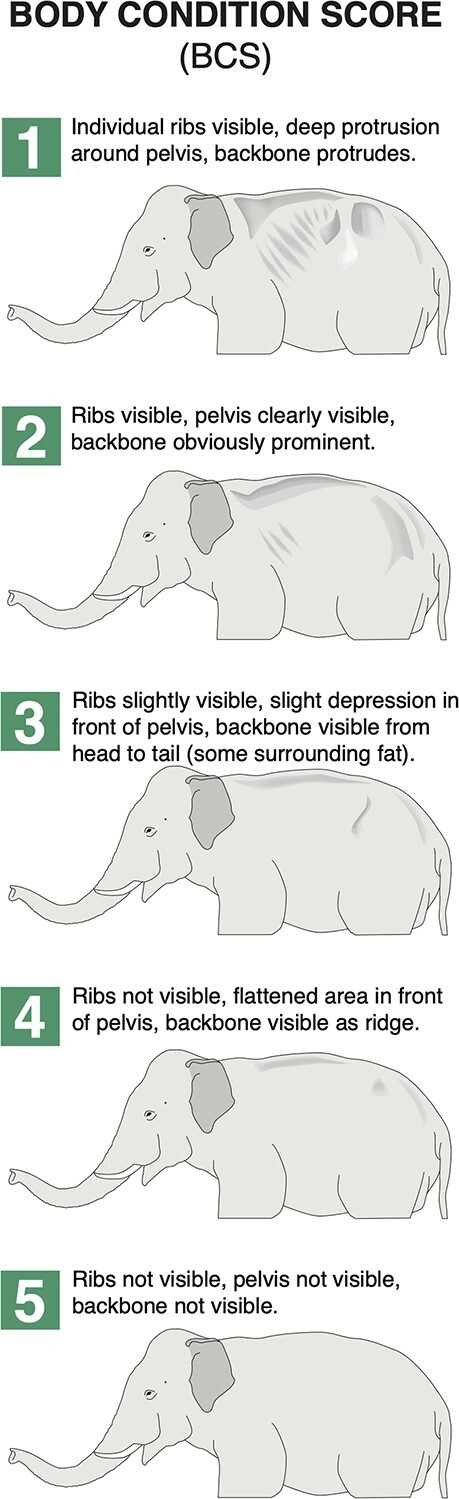
Visual scale used by zoo staff to record BCS upon collection of each fecal sample. BCS was recorded from 1 (underweight) to 3 (ideal weight) to 5 (overweight) after inspection of ribs, pelvis, and backbone, based on descriptions from Pokharel *et al.* (2017). Illustrations by C. LaDue.

Upon collection of each sample, zoo staff noted any presence of visible indicators that are characteristic of musth in Asian elephants [temporal gland secretions (TGS) and/or urine dribbling (UD)] using visual scales described and validated in LaDue *et al.* (2022c) and LaDue *et al.* (2022a). These indicators were used to help define musth status, described below. Staff also recorded the elephant’s body condition score (BCS) [from a score of one for underweight animals to a score of five for overweight animals, using standards from Pokharel *et al.,* 2017, (Figure 1)] at the time of defecation. As a proxy for social exposure, staff indicated the amount of time (rounded to the nearest whole hour) that the elephant spent within potential tactile proximity with male and female conspecific(s) [e.g. sharing the same space, or in a directly adjacent space permitting tactile interactions; indicated separately for time spent with male(s) and time spent with female(s)] within the 24 h prior to sample collection. This measure of social exposure reflects the presumed ~ 24 h lag between hormone release and fecal excretion in elephants (Ganswindt *et al.,* 2003; Fuller *et al.,* 2011).

### 2.3. Hormone metabolite extraction

The detailed extraction protocol used in this study is provided in LaDue (2022) and LaDue *et al.* (2022a) and was based on those commonly used to analyze fecal steroid and thyroid hormone metabolites in elephants and other mammals (Ganswindt *et al.,* 2005; Wasser *et al.,* 2010; Pokharel *et al.,* 2017; Szott *et al.,* 2020a). Briefly, fecal samples were dried at 55°C in a laboratory drying oven (Thermolyne model F30438CM; Thermo Scientific; Asheville, NC) for about 72 h until all moisture had evaporated. Then, approximately 0.2500 g of sifted and mixed fecal powder (weighed to the nearest 0.0001 g on a digital laboratory scale, Entris II BCE64I-1S; Sartorius Lab Instruments GmbH; Goettingen, Germany) was placed in a borosilicate glass tube (Fisher Scientific; Pittsburgh, PA) 5.00 mL of 100% methanol was added to each tube and mixed for 30 min on a large capacity mixer (Glas-Col; Terre Haute, IN; speed ≈1000 rpm). Tubes were centrifuged for 5 min at 935 *g* (Thermo Scientific Sorvall ST Plus Series Centrifuge; Thermo Fisher Scientific; Waltham, MA), and then 2.00 mL of supernatant was recovered and transferred to a clean borosilicate glass tube (Fisher Scientific; Pittsburgh, PA). Supernatants were dried using a vacuum concentrator (Savant SpeedVac SPD1030; Thermo Fisher Scientific; Waltham, MA) at 45°C for 75 min. Extracted samples were reconstituted in 500 μL of assay buffer (buffer X065; Arbor Assays; Ann Arbor, MI), vortexed for 5 sec each (Vortex Genie 2; Scientific Industries; Bohemia, NY), and finally sonicated for 5 min (Branson M3800 Ultrasonic Cleaner; Emerson Electric; St. Louis, MO). Each resulting fecal extract was transferred to a polypropylene tube (Perfector Scientific; Atascadero, CA) and stored at—20°C until dilution and analysis. All assays occurred within three months of extraction.

### 2.4. Enzyme immunoassay analysis

Fecal extracts were analyzed for FAM, FGM, and FT3 via double-antibody enzyme immunoassay (EIA) using commercially available kits (Arbor Assays; Ann Arbor, MI): testosterone, catalog no. K032; corticosterone, catalog no. K014; and T3, catalog no. K056. We precisely followed the manufacturer’s protocols for testosterone, corticosterone, and T3 EIAs. All full-strength (1:1) extracts were diluted to 1:49 for testosterone and 1:8 for corticosterone and T3 based on parallelism validation results; these dilutions fell near 50% bound for each assay, the area of greatest assay precision.

Alongside fecal extracts, we also assayed a control in each assay plate consisting of a pooled sample at 1:128 for FAM and 1:16 for FGM and FT3. We included a full standard curve in all assays, and standards (including blank and non-specific binding wells), samples, and controls were run in duplicate. Samples were assayed in a pseudorandomized order for each hormone using a random number generator to minimize potential influences of intra- and inter-assay variation. Optical density of each well was read at 450 nm using a microplate reader (Epoch Microplate Spectrophotometer; Bio Tek Instruments; Winooski, VT), and metabolite concentration was calculated using a sigmoidal dose response curve in Prism version 9.3 (GraphPad; San Diego, CA). FAM, FGM, and FT3 concentrations are reported as ng/g of dried feces, corrected for volumetric differences during the extraction process. Any samples with < 10% or > 90% binding or with coefficients of variation (CVs) >10% were reanalyzed (re-diluting as necessary when binding was too high or low). Intra-assay variation was < 10% for all assays in this study; the manufacturer provided average intra-assay precision values of 10.9% for testosterone, 5.2% for corticosterone, and 6.3% for T3. The inter-assay CV for the controls was 3.9% for testosterone (*n* = 48), 4.5% for corticosterone (*n* = 50), and 3.1% for T3 (*n* = 23).

Because FT3 concentrations remain relatively stable in male elephants over time (Brown *et al.,* 2007) and to reduce laboratory costs, we analyzed monthly instead of weekly samples for FT3 for each elephant while he was not showing visible indicators of musth (i.e. TGS and/or UD). However, because we hypothesized that FT3 concentration would vary around musth, we analyzed weekly samples for FT3 for each male while he was exhibiting TGS and/or UD. Therefore, models with FT3 included as a response variable (described below) include only *n* = 595 samples.

#### 2.4.1. Assay validation

We first performed a parallelism validation for each hormone assay consisting of 12 serial dilutions of pooled fecal extract assayed alongside known standards; each assay showed good parallelism with slopes not significantly different from those of the standard curves: testosterone (*F*_1,11_ = 0.315, *P* = 0.586), corticosterone (*F*_1,8_ = 2.763, *P* = 0.135) and T3 (*F*_1,10_ = 2.576, *P* = 0.140) (LaDue, 2022). Subsequently, we further validated assays with accuracy tests (matrix effect tests) by spiking standard curves of each hormone with equal volumes of low-hormone-concentration fecal extract pool at 1:49 dilution for testosterone and 1:8 dilution for corticosterone and T3, assaying these along standards spiked with assay buffer. All three assays demonstrated good accuracy upon inspection of slopes, intercepts, and linearity (LaDue, 2022): testosterone, slope = 1.104, intercept = 118 pg/mL, pool = 57 pg/mL, *R* = 1.0000; corticosterone, slope = 1.008, intercept = 118 pg/mL, pool = 104 pg/mL, *R* = 0.9997; T3, slope = 1.242, intercept = 215 pg/mL, pool = 127 pg/mL, *R* = 0.9996. Biological validations of each assay for Asian elephant fecal samples are described in LaDue (2022) and LaDue *et al.* (2022a). Logistical challenges prevented us from performing physiological validations such as ACTH challenges, but such validations have been performed successfully for elephants in prior studies (Ganswindt *et al.,* 2003; Santymire *et al.,* 2012) and fecal hormone literature generally shows robust ability of fecal hormone assays to detect physiologically induced elevations of steroid hormones across mammalian taxa (e.g. Dloniak *et al.,* 2004; Hogan *et al.,* 2010; Pribbenow *et al.,* 2015; Pribbenow *et al.,* 2016; Kozlowski *et al.,* 2020; Mondol *et al.,* 2020).

### 2.5. Defining a musth episode

The onset and duration of a musth episode can be defined by changes in a male elephant’s androgen concentrations and the subsequent onset of visible musth indicators (TGS and/or UD) (Jainudeen *et al.,* 1972a; Brown *et al.,* 2007; Chave *et al.,* 2019). We used a stepwise process to identify “true” or “confirmed” musth episodes [i.e. episodes that include prolonged elevations in testosterone combined with visible TGS and/or UD, as described in the literature (Jainudeen *et al.,* 1972a; Brown *et al.,* 2007)]. For each elephant in this study, we calculated a baseline FAM value using an iterative process (Brown *et al.,* 1996) in R version 4.1.0 (R Core Team, 2021) with the package *hormLong* (Fanson and Fanson, 2015). In each iteration, all FAM concentrations exceeding the mean plus 1.5 times the standard deviation were removed [mean_FAM_ + (1.5 × SD_FAM_)], and this process continued until no values exceeded this criterion. The remaining FAM values for each elephant described that individual’s baseline, and the maximum baseline FAM concentration defined the upper baseline threshold (UBT) value.

**Figure 2:**
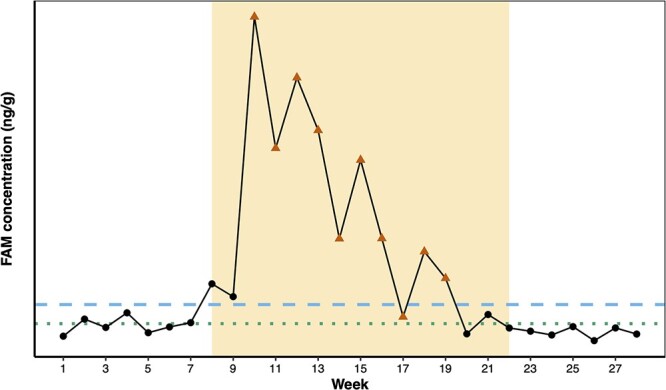
Example FAM profile demonstrating the parameters used to define a confirmed musth episode among Asian elephants in this study. Each point represents a sample, with the yellow shaded region defining the duration of a confirmed musth episode. Blue dashed line represents the UBT (the maximum FAM baseline value that remained within the range of iterative calculations of the overall mean FAM concentration plus 1.5 times the standard deviation), and green dotted line is the LBT (calculated by averaging the baseline FAM values and adding two times the baseline standard deviation). Points marked with red triangles indicate samples collected when a male elephant was exhibiting visible musth indicators (temporal gland secretions and/or urine dribbling).

For each elephant, the onset of a putative musth episode was designated as the first date when the FAM concentration exceeded the UBT (Figure 2). After that, we followed standards described by Chave *et al.* (2019) to define the duration of a putative musth episode, with the following criteria: (1) the episode was sustained as long as the male’s FAM concentration remained above the lower baseline threshold (LBT), defined as the mean FAM baseline plus two times the baseline standard deviation [mean_baseline_ + (2 × SD_baseline_)]; and (2) the episode ended with two consecutive weeks below the LBT (the last day of the episode was the first day it dropped below the LBT). Single-point deviations above or below the UBT or LBT were first assigned to be musth or non-musth based on the surrounding data points. Further, to account for regular androgen fluctuations around baseline values, if no TGS or UD was observed over the study period (i.e. TGS = 0 or 1, and UD = 0 across all samples), then we concluded that an elephant had not entered musth over the study period (*n* = 8 elephants).

After applying the above criteria, we identified 57 putative musth episodes in 18 elephants. We excluded 19 of these in which the male’s FAM concentration remained above the UBT but in which there were no accompanying external indicators of musth (i.e. TGS or UD) within one month of the beginning or end of the episode. Instead of classifying these as musth episodes, we designated them “elevated FAM episodes” for separate analysis. In male Asian elephants, elevated androgen fluctuations may occur around puberty that are distinct from fully mature musth episodes (Rasmussen *et al.,* 2002) or that result from sexual stimulation (e.g. presence of estrous female or other nearby sexual activity); similarly, Poole *et al.* (1984) reported elevated androgen levels in free-ranging African savanna elephants (*Loxodonta africana*) comparable to musth but without any external musth indicators. Prolonged shifts around baseline also may occur with fecal hormone metabolites (Millspaugh and Washburn, 2004). After excluding these, we were left with 38 putative musth episodes in 18 elephants (i.e. elevated FAM that was also accompanied by at least one external musth indicator). We further eliminated six of these episodes whose highest peak FAM values did not exceed the highest peak FAM concentration of any of the elevated FAM episodes (lowest peak concentration = 367.48 ng/g). This resulted in 32 confirmed musth episodes in 17 elephants, and 19 elevated FAM episodes in 11 elephants.

### 2.6. Statistical analysis

Analyses were conducted in R version 4.1.0 (R Core Team, 2021) with the package *tidyverse* (Wickham *et al.,* 2019); for all analyses involving *P*-value-based hypothesis testing, statistical significance was set at *α* = 0.05. We calculated the mean, minimum, maximum, and standard deviation of FAM, FGM, and FT3 values separately for elephants that did (*n* = 17) and did not (*n* = 9) exhibit confirmed musth episodes over the study period, and separately for samples from confirmed musth episodes, elevated FAM episodes and non-musth periods. When calculating these descriptive statistics, data were pooled among male elephants beforehand to account for uneven sampling between individuals. We investigated significant differences between musth groups (those elephants that underwent a confirmed musth episode versus those that did not) and musth status categories (non-musth, confirmed musth, elevated FAM) in average concentrations of each hormone metabolite with linear mixed models (LMMs) using restricted maximum likelihood. This approach allowed us to account for repeated, uneven sampling from the same individual elephants and facilities—these variables were included as random factors in the models.

#### 2.6.1. The influence of androgens on FGM and FT3 concentrations

After visual inspection with quantile-quantile (Q-Q) plots revealed non-normal distributions in FAM, FGM, and FT3 concentrations, we log_10_-transformed each of these measures to improve the distribution of these data. We constructed LMMs using restricted maximum likelihood for FGM and FT3 to investigate a potential correlation of either hormone with FAM. For each hormone, we constructed separate models for each group (musth and no-musth), and all models included animal identity as a random effect.

#### 2.6.2. Comparison of elevated FAM episodes versus confirmed musth episodes

To investigate potential differences between elevated FAM episodes versus confirmed musth episodes, we compared duration of the episode, peak (maximum) FAM concentration, age of the elephant, and average BCS between elevated FAM episodes and confirmed musth episodes, with LMMs using restricted maximum likelihood (including animal identity and facility as random factors). Duration of an episode was conservatively calculated via subtraction of the day of the first elevated FAM sample from the day of the last elevated FAM sample. Because of the long duration of musth episodes and relatively short study period, we could not calculate accurate frequencies (e.g. average number of episodes per year) of elevated FAM or musth episodes. Due to our small sample sizes (*n* = 19 elevated FAM episodes; *n* = 32 confirmed musth episodes), we used the package *afex* (Singmann *et al.,* 2021) to evaluate the statistical significance of fixed effects using Kenward-Roger approximations that minimize Type I error (Luke, 2017). Similarly, to characterize variation in the duration of elevated FAM episodes and confirmed musth episodes (separately), we used the same LMM approach. We included the following fixed effects in each model: age at beginning of episode, average BCS throughout episode, average social exposure to male conspecifics, and average social exposure to female conspecifics. Animal identity and facility were included in both models as random factors to account for repeated episodes at the same facilities.

#### 2.6.3. Factors influencing variation in fecal hormone metabolite concentrations

We evaluated the influence of various factors on log_10_-transformed hormone metabolite concentrations with LMMs guided by an information theory approach (Burnham and Anderson, 2002; Johnson and Omland, 2004; Zuur and Ieno, 2016). We constructed separate models for each of the three hormones (FAM, FGM, and FT3) using the package *lme4* (Bates *et al.,* 2015). For each hormone/group, we constructed a list of candidate models that included the following fixed effects in various combinations: age at fecal sample collection, BCS, the number of males at the facility, the number of females at the facility, social exposure to males over the previous 24 hr, and social exposure to females over the previous 24 hr (Table A1). In all models, elephant and facility identity was included as a random effect. For FGM and FT3, we also included musth status (non-musth, elevated FAM, or musth, determined by FAM concentration) in all candidate models. Candidate models were ranked via Akaike information criteria (AIC) with maximum likelihood estimation (Burnham and Anderson, 2002) using the packages *AICcmodavg* (Mazerolle, 2019) and *MuMIn* (Bartón, 2019). Each of the highest ranked models then underwent a process to exclude non-significant variables (*P* > 0.05) with a modified *χ*^2^ test using restricted maximum likelihood. For each of these models, we estimated the explanatory value for predicting the respective hormone metabolite concentration using a marginal coefficient of variation (*R*^2^*_c_*). We confirmed none of the factors in the final models were collinear using the variation inflation factor (VIF) (Dormann *et al.,* 2013); all VIF values were less than 2.

## 3. Results

**Figure 3:**
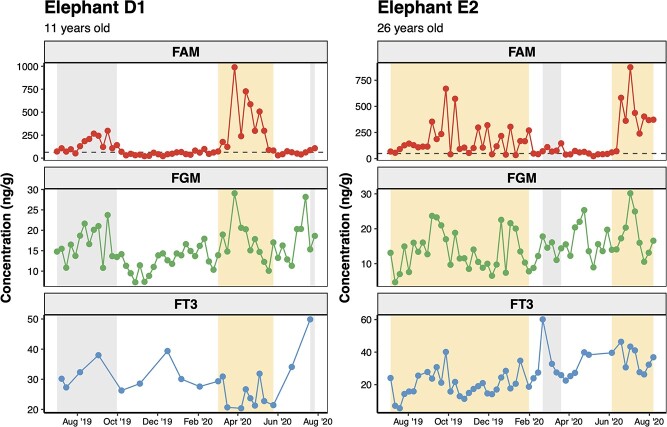
Individual hormone profiles of representative male Asian elephants “D1” and “E2,” showing longitudinal concentrations of FAMs, FGMs and FT3. Circles represent individual fecal samples with solid lines connecting consecutive samples; orange shaded regions indicate confirmed musth episodes, and gray shaded regions show elevated FAM episodes. The UBT of FAM for each elephant is shown with the dashed line in the FAM panels.

Representative hormone profiles are presented in Figure 3; refer to LaDue (2022) for profiles of all elephants included in the study. Overall FAM concentrations were significantly different between the elephants that exhibited at least one confirmed musth episode (termed “confirmed-musth elephants,” *n* = 17) and those that did not (termed “no-musth elephants,” *n* = 9) (*F*_1, 24.12_ = 8.36, *P* = 0.008), but FGM was not significantly different between these two groups (*F*_1, 19.42_ = 2.23, *P* = 0.152) or FT3 (*F*_1, 24.09_ = 0.01, *P* = 0.935) Table 2. Further comparisons of non-musth samples between the confirmed-musth and no-musth groups did not reveal significant differences in the average concentration of any of the hormone metabolites we tested (FAM: *F*_1, 18.50_ = 0.30, *P* = 0.590; FGM: *F*_1, 18.91_ = 0.00, *P* = 0.975; FT3: *F*_1, 23.02_ = 0.03, *P* = 0.859). Within the confirmed-musth elephants, average FAM and FGM concentrations were significantly higher during elevated FAM and musth episodes compared to non-musth (FAM: *F*_1, 877.18_ = 89.02, *P* < 0.001; FGM: *F*_1, 446.51_ = 14.51, *P* < 0.001), but FT3 concentrations remained unchanged across these periods (*F*_1, 891.69_ = 2.08, *P* = 0.126). Likewise, we did not find any significant differences between non-musth and elevated FAM episodes in the “no-musth” elephants (FAM: *F*_1, 406.66_ = 1.57, *P* = 0.211; FGM: *F*_1, 402.56_ = 0.03, *P* = 0.869; FT3: *F*_1, 400.94_ = 0.84, *P* = 0.359).

**Table 2:** Descriptive statistics (mean, minimum, maximum and standard error) for FAM, FGM and FT3 concentrations in male Asian elephants included in the study, reported in ng/g of dried feces. Before obtaining these statistics, pooled means, minimums, maximums and standard errors were calculated for each elephant to account for uneven sampling. Males are divided into two groups: those that had at least one confirmed musth episode over the study period (confirmed-musth elephants, n = 17 elephants) and those that did not (no-musth elephants, n = 9 elephants). Additionally, samples are divided into three categories: non-musth samples, samples collected during elevated FAM episodes, and samples collected during confirmed musth episodes (see text for category definitions). Sample size (n) indicates number of samples collected in each category (before pooling among elephants) for FAM and FGM (reduced number of samples was analyzed for FT3; see text for details). SE = standard error.

		FAM				FGM				FT3			
	*n*	Mean	Min.	Max.	SE	Mean	Min.	Max.	SE	Mean	Min.	Max.	SE
**Confirmed-musth elephants**	**896**	**198.77** ^a^	**10.38**	**4063.20**	**35.92**	**16.82**	**4.68**	**60.76**	**1.11**	**35.64**	**3.31**	**106.05**	**2.78**
Non-musth	378	58.35^b^	10.38	566.18	6.72	15.97^b^	4.77	57.84	1.05	33.35	9.84	87.77	2.37
Elevated FAM episodes	100	105.31^b^	28.14	367.48	14.34	15.94^b^	6.39	37.07	1.14	35.02	16.99	76.08	3.31
Musth episodes	418	394.17^b^	21.38	4063.20	77.81	18.20^b^	4.68	60.76	1.15	38.21	3.31	106.05	2.95
													
**No-musth elephants**	**413**	**83.78** ^a^	**7.29**	**1914.62**	**12.81**	**15.39**	**4.96**	**52.17**	**0.82**	**35.01**	**8.25**	**118.56**	**2.09**
Non-musth	411	78.44	7.29	1914.62	12.52	15.68	4.96	52.17	0.67	35.83	8.25	118.56	2.06
Elevated FAM episodes	2	131.86	116.43	147.28	15.42	12.84	10.63	15.04	2.20	27.58	27.58	60.11	7.64

a Significant difference (*P* < 0.05) between elephants that exhibited a confirmed musth (“confirmed-musth elephants”) and those that did not (“no-musth elephants”).

b Significant difference (*P* < 0.05) within the confirmed-musth elephants between musth statuses (non-musth, elevated FAM, musth).

### 3.1. The influence of androgens on FGM and FT3 concentrations

FGM concentrations (log_10_-transformed) were positively correlated with log_10_(FAM) in both the “musth” group (model coefficient = 0.124, *F*_1, 832.68_ = 178.63, *P* < 0.001) and the “no-musth” group (model coefficient = 0.199, *F*_1, 395.32_ = 59.67, *P* < 0.001). Similarly, we found positive correlations between log_10_(FT3) and log_10_(FAM) in both the musth group (model coefficient = 0.050, *F*_1, 825.06_ = 14.80, *P* < 0.001) and the no-musth group (model coefficient = 0.076, *F*_1, 394.45_ = 4.02, *P* = 0.046).

### 3.2. Comparison of elevated FAM episodes versus confirmed musth episodes

Elevated FAM episodes lasted on average ± SE 31.47 ± 5.12 days (min = 7 days, max = 91 days), while confirmed musth episodes lasted significantly longer, 91.59 ± 10.43 days (min = 22 days, max = 210 days) (*F*_1, 47.98_ = 18.74, *P* < 0.001). Further, peak FAM concentrations were higher in musth episodes (1342.64 ± 171.06 ng/g) compared to elevated FAM episodes (161.60 ± 22.68 ng/g) (*F*_1, 31.46_ = 7.14, *P* = 0.012). While elephants exhibiting confirmed musth episodes tended to be older than those undergoing elevated FAM episodes (average of 32.17 ± 2.59 years and 26.59 ± 3.70 years, respectively), this difference was not statistically significant (*F*_1, 7.91_ < 0.01, *P* = 0.997). The youngest elephant that exhibited an elevated FAM episode was 10.74 years old; the same elephant exhibited a confirmed musth episode approximately two months after that elevated FAM episode at 11.08 years of age (the youngest age at which we recorded musth in our study population). There was no difference in BCS between elephants displaying elevated FAM episodes (3.77 ± 0.22) versus those during confirmed musth episodes (3.89 ± 0.17) (*F*_1, 2.54_ = 0.13, *P* = 0.749).

**Figure 4:**
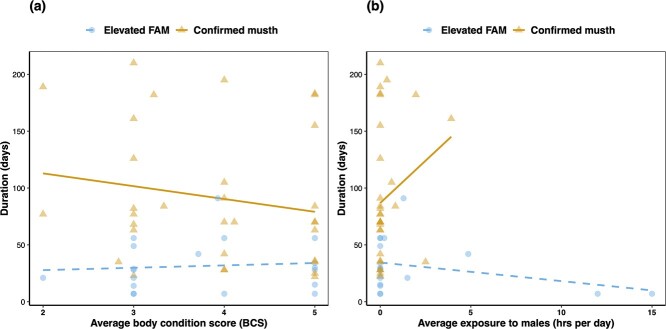
Association between duration of elevated FAM and confirmed musth episodes in male Asian elephants (*n* = 26) and **(a)** average BCS and **(b)** average exposure to male conspecifics. Blue circles represent individual elevated FAM episodes, and orange triangles indicate individual confirmed musth episodes. Dashed blue lines and solid orange lines show linear regressions for elevated FAM episodes and confirmed musth episodes, respectively. BCS values were significantly associated with duration for confirmed musth episodes (*P* = 0.045) but not elevated musth episodes (*P* = 0.536). However, the duration of both elevated FAM episodes (*P* = 0.029) and confirmed musth episodes (*P* = 0.033) differed significantly with exposure to male conspecifics.

We found that neither age (*F*_1, 1.87_ = 7.12, *P* = 0.125), BCS (*F*_1, 6.65_ = 0.63, *P* = 0.455), nor social exposure to females (*F*_1, 4.64_ = 0.15, *P* = 0.718) influenced the duration of elevated FAM episodes; however, social exposure to males significantly influenced the duration of these episodes (*F*_1, 13.20_ = 7.07, *P* = 0.019), with longer average exposure to male conspecifics associated with shorter episodes (Figure 4). However, the duration of confirmed musth episodes was significantly associated with both BCS (*F*_1, 15.36_ = 4.75, *P* = 0.045) and social exposure to males (*F*_1, 25.97_ = 5.08, *P* = 0.033) (Figure 4). Higher average BCS values predicted shorter musth periods, while longer average exposure to male conspecifics resulted in more prolonged musth periods. Musth duration was not associated with age (*F*_1, 12.98_ = 0.16, *P* = 0.700) or social exposure to females (*F*_1, 13.57_ = 0.48, *P* = 0.501).

### 3.3. Factors influencing variation in fecal hormone metabolite concentrations

**Figure 5:**
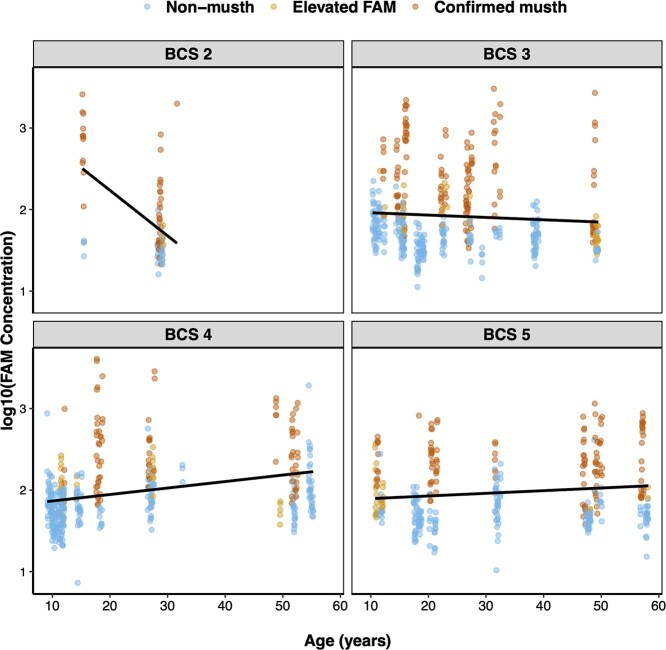
Variation of FAM concentrations in male Asian elephants (*n* = 26) in relation to age, BCS and musth status (non-musth, elevated FAM episode, confirmed musth episode). Points represent individual fecal samples, and lines show linear model prediction for each BCS score.

**Figure 6:**
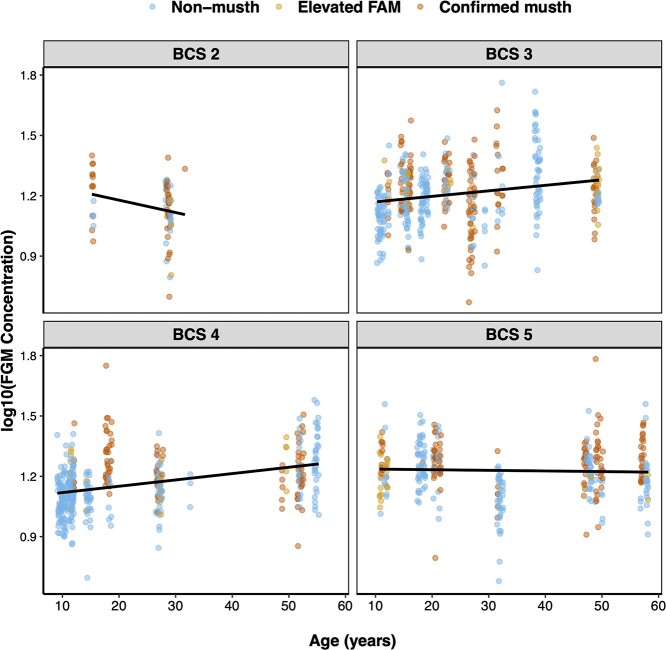
Variation of FGM concentrations in male Asian elephants (*n* = 26) in relation to age, BCS and musth status (non-musth, elevated FAM episode, confirmed musth episode). Points represent individual fecal samples, and lines show linear model prediction for each BCS score.

We found a variety of factors that influenced hormone metabolite concentrations (Table 3, Table A2). FAM concentrations were influenced by the interaction between age and BCS, the exposure to male conspecifics, and the interaction between the number of and exposure to female conspecifics (*R*^2^*_c_* = 0.346). Specifically, age was directly related to FAM concentration when BCS = 4 or 5, but age showed an inverse relationship with FAM concentrations when BCS = 2 or 3 (Figure 5). Additionally, increased exposure to adult males at the facility was associated with decreased FAM concentrations, and the interaction between the number of and exposure to adult females increased FAM concentrations (generally, concentrations were highest in cases in which there were a high number of female conspecifics and males experienced prolonged exposure to those females). We also found that higher FGM concentrations were associated with both elevated FAM episodes and confirmed musth episodes, as well as the interaction between age and BCS (*R*^2^*_c_* = 0.299) (Figure 6), with similar trends observed as with FAMs (e.g. FGM concentrations increased with age, except when BCS = 2). Finally, while we did not identify factors that contributed to variation in FT3 concentrations, we found that the null model that incorporated differences between individual elephants accounted for much of the variation in FT3 (*R*^2^*_c_* = 0.439).

**Table 3:** Model estimates of LMM analysis for log_10_-transformed concentrations of FAMs, FGMs and FT3s in male Asian elephants. Back-transformed estimates are reported in ng/g of dried feces. SE = standard error. Refer to Table A1 for parameter definitions and reference values. ♂ = male, ♀ = female.

	Fixed effect	Estimate	SE	*t*-value
**log**_**10**_ **(FAM)**	Intercept	2.411	0.310	7.788
	Age	−0.005	0.013	−0.348
	BCS 3	−0.082	0.279	−0.293
	BCS 4	−0.470	0.298	−1.578
	BCS 5	−0.196	0.363	−0.540
	♂ exposure	−0.010	0.003	−4.048
	♀ conspecifics	−0.012	0.007	−1.775
	♀ exposure	−0.011	0.004	−2.668
	Age: BCS 3	−0.007	0.013	−0.526
	Age: BCS 4	0.012	0.013	0.919
	Age: BCS 5	0.004	0.014	0.299
	♀ conspecifics: ♀ exposure	0.002	0.001	2.235
**log**_**10**_ **(FGM)**	Intercept	1.145	0.092	12.482
	Musth status (elevated FAM)	0.026	0.015	1.659
	Musth status (musth)	0.054	0.010	5.696
	Age	0.002	0.004	0.481
	BCS 3	−0.010	0.087	−0.119
	BCS 4	−0.053	0.090	−0.593
	BCS 5	0.051	0.095	0.535
	Age: BCS 3	<0.001	0.004	0.085
	Age: BCS 4	0.001	0.004	0.221
	Age: BCS 5	−0.003	0.004	−0.642
**log**_**10**_ **(FT3)**	Intercept	1.462	0.044	32.980

## 4. Discussion

In this study, we demonstrated that male Asian elephants exhibit considerable variation in musth and its endocrine correlates, as measured non-invasively through feces (i.e. fecal androgen, glucocorticoid, and T3 metabolites), and that this variation can at least partly be explained by several factors including age, body condition, and exposure to conspecifics of both sexes. First, we found that FAM and FGM concentrations—but not FT3 concentrations—were significantly higher during musth and elevated FAM episodes. As FAMs were used to define musth episodes, high FAM concentrations during these periods are unsurprising, but it is interesting to note that several factors influenced these FAM concentrations. For instance, FAM concentrations were negatively associated with exposure to male conspecifics, but positively associated with the positive interaction between the number of and exposure to female conspecifics (i.e. FAM was higher if males had more consistent exposure to multiple females). Additionally, FAM concentrations increased with age at BCS values of 4 or 5 (which are considered overweight), but decreased with age at BCS values of 2 (underweight) or 3 (ideal weight). Similarly, we found that the interaction between age and BCS influenced FGM concentrations, with a direct relationship between age and FGM concentration at BCS = 3 or 4, and the opposite relationship with age at BCS = 5. We found no extrinsic or intrinsic factors that influenced FT3 concentrations, but a large proportion of the variation in FT3 could be attributed to differences between individual elephants and facilities. In comparing confirmed musth and elevated FAM episodes, we found that musth episodes were significantly longer and peak FAM concentrations were higher, but there was no significant difference in the age or BCS of males undergoing either of these episodes. However, exposure to male conspecifics was associated with shorter duration of elevated FAM episodes and, conversely, with longer duration of confirmed musth episodes. Additionally, males with higher BCS values had shorter musth episodes.

Several males of prime reproductive age [e.g. those between 20 and 50 years of age (Sukumar, 1989)] failed to exhibit confirmed musth episodes over the study period, despite access to male and female conspecifics and/or ample food. Extending our sampling period beyond 12 months may have indicated that these “no-musth” males are actually capable of undergoing musth, but even so, studies of Asian elephants in range countries indicate that musth should occur annually (Lincoln and Ratnasooriya, 1996; Sukumar, 2003). In the better-studied African savanna elephant, social pressures and physiological status can affect the ability of a male to exhibit musth (Poole, 1987; Poole, 1989; Ganswindt *et al.,* 2005), and presumably similar factors influence musth in *E. maximus* (Rasmussen and Perrin, 1999; Schulte and Rasmussen, 1999). Indeed, we found that FAM concentration was positively associated with age at high body conditions (BCS = 4 or 5), but negatively associated with age at low body conditions (BCS = 2 or 3). That is, those older elephants with less fat reserves tended to have lower FAM concentrations. However, the duration of musth episodes was negatively associated with body condition (i.e. shorter musth episodes if fat reserves are higher), perhaps indicating that excessive fat stores impede on a male’s ability to sustain a heightened sexual state, as has been reported in zoo-housed female African elephants (Freeman *et al.,* 2009; Morfeld and Brown, 2014). Conventionally, animal caregivers have occasionally reduced the diets of their male elephants in an attempt to reduce the duration of excessively long periods of musth (Jainudeen *et al.,* 1972b; Cooper *et al.,* 1990; Lincoln and Ratnasooriya, 1996; Olson, 2004). Musth is thought to be energetically expensive ([Bibr ref112][Bibr ref112]; Dickerman *et al.,* 1994; Rasmussen and Perrin, 1999), and several studies have reported that the musth cycles of older dominant elephants are distinct from those of younger males (e.g. in terms of frequency, duration, and/or intensity) (Lincoln and Ratnasooriya, 1996; Ganswindt *et al.,* 2010); our results support these explanations. Interestingly, we also found evidence of social influences on FAM concentrations. FAM concentrations decreased as exposure to male conspecifics increased (suggesting that musth may be suppressed by the presence of male conspecifics), but FAM concentrations increased with the positive interaction of the number of and exposure to female conspecifics (indicating that interactions with females promotes sexual activity). Unexpectedly, however, confirmed musth episodes were longer in those males exposed to other male elephants. While this result may seem to contradict the effect of male conspecifics on FAM concentrations, it is possible that the presence of male conspecifics suppresses musth until a favorable physiological and/or socioecological state is reached, at which point consistent exposure to other male elephants stimulates a male to remain in musth as long as is feasible to gain more mating opportunities. Further, males in musth may acquire enhanced dominance status, and therefore it may be worthwhile to prolong musth to maintain that status over conspecifics (Lincoln and Ratnasooriya, 1996; Chelliah and Sukumar, 2013; Keerthipriya *et al.,* 2020). Other studies have suggested a variety of factors that may influence suppression or incitement of musth among male elephants (Poole, 1987; Poole, 1989; Brown *et al.,* 2007; Chave *et al.,* 2019; Glaeser *et al.,* 2022), but this study offers systematic evidence that the interaction between physiological (e.g. age, body condition) and social variables is associated with variation in androgen secretion. This variation could in part explain why some of the males in our study did not exhibit musth, although certainly individual differences between elephants also influence these patterns. We suggest that future studies are warranted to more carefully characterize the influence of these factors on the occurrence of musth, including diet, health condition, sexual status of male and female conspecifics (e.g. musth vs. non-musth, follicular vs. luteal), and potential behavioral mechanisms (e.g. dominance interactions) for the suppression of musth.

Our results also suggest that musth in zoo-housed Asian elephants is associated with increased HPA activity: FGM concentrations were elevated in confirmed musth and elevated FAM samples compared to non-musth samples. Furthermore, FGM concentrations were positively correlated with FAM concentrations, supporting others’ findings from zoo-housed Asian elephants that indicate musth is a physiologically stressful state (Brown *et al.,* 2007; Chave *et al.,* 2019). Our previous work comparing zoo-housed to wild elephants suggest this relationship between FGM and FAM may only occur in *ex-situ* populations where nutrition and environmental influences are quite different from *in-situ* populations (LaDue *et al.,* 2022a). Androgens and glucocorticoids are commonly associated in male vertebrates, as the often complex factors involved in locating, attracting, and/or breeding with a receptive mate can be stressful for males (Wingfield and Sapolsky, 2003). Aside from musth status, we also found that age and body condition interacted to predict variation in FGM concentrations; FGM concentrations increased with age when BCS = 2, 3, or 4, but decreased with age when BCS = 5. Further, when analyzing the main effect of body condition separately from age, FGM concentrations were lower at intermediate BCS values (3 or 4) compared to BCS values at either extreme (2 or 5). Increased glucocorticoid concentrations with age have previously been reported in zoo-housed male elephants (Brown *et al.,* 2007) and may be explained by the increased physiological cost associated with increased dominance in older animals (Sapolsky, 2005; Gobush *et al.,* 2008) [but see Barrette *et al.,* 2012 and Levy *et al.,* 2020] and/or normal aging processes (Sapolsky *et al.,* 1986; Sapolsky, 1991). Additionally, environmental variables (e.g. season) have been reported to affect the relationship between BCS and glucocorticoids in free-ranging Asian elephants (Pokharel *et al.,* 2017). As we found that FGMs were positively related to FAM levels, it is reasonable that these same factors could also influence musth.

We did not find that FT3 concentrations were related to musth in this study. Variation in FT3 was not associated with any other variable we measured either, save for a positive correlation with FAM. Previous studies have documented decreased concentration in circulating T3 and T4 in serum around musth (Brown *et al.,* 2007; Chave *et al.,* 2019), so we expected that FT3 would increase during musth to reflect the transition to a sexually active, metabolically taxing state. We also predicted that FT3 concentrations would fluctuate in response to changes in body condition (as a proxy for metabolic status) as has been reported in free-ranging African elephants (Szott *et al.,* 2020a). However, 18 of 26 elephants in our study did not change BCS values over the study period, and only two of these elephants exhibited decreased body condition during musth, likely because they always had consistent access to high-quality food. Thus, low intra-individual variation in BCS may have hindered our ability to detect relationships between BCS and FT3. Still, while our models did not identify environmental or social factors influencing FT3, they did identify substantial FT3 variation between individuals and/or elephant facilities. Aside from confirming the association of thyroid hormones with musth, future studies should also investigate differences in nutrition and health that may also influence thyroid hormone activity in this species.

The identification of at least some factors that contribute to hormonal variation around musth complements the findings of other studies of elephant health and wellbeing to have strong implications for zoo elephant welfare. For instance, while the behavioral benefits of socializing male elephants have been described (Williams *et al.,* 2019; Schreier *et al.,* 2021; Readyhough *et al.,* 2022; LaDue *et al.,* 2022d), our study is one of the first to systematically demonstrate the physiological correlates of male socialization in Asian elephants (but see Moresco *et al.,* 2022). During musth, Asian elephants in the wild are typically more social than when they are not in musth, especially at older ages (Keerthipriya *et al.,* 2020; LaDue *et al.,* 2022d). However, zoos often separate musth males from other elephants as a precaution against potential aggression. Here, we show that the social environment likely impacts how musth is manifested, and so beyond implications for reproduction, there are also welfare considerations to be weighed, as a male’s social needs may also change during musth. We have also shown that a physical indicator of welfare (i.e. body condition) is associated with altered hormonal activity in male Asian elephants. While most of the elephants in our sample did not experience changes in their body condition across the study period due to musth, our results suggest that a male’s body condition is associated with physiological correlates of musth. Obesity is a common concern in zoo-housed elephants (Morfeld *et al.,* 2016; Schiffmann *et al.,* 2018), and further, body condition (as a proxy for adiposity) may also reflect metabolic functioning in zoo-housed Asian elephants (Chusyd *et al.,* 2021). Elephant husbandry practices—such as exercise and feeding—have been linked to body condition (Morfeld *et al.,* 2016), and so male elephants may be sensitive to low levels of activity and high-calorie diets. As stated previously, higher glucocorticoids were also associated with over-conditioned and under-conditioned male elephants (regardless of musth status) in our study. Therefore, high or low body fat may negatively impact a male elephant’s ability to cope with physiological and/or environmental stressors, a potential indicator of compromised welfare. Finally, a modern understanding of animal welfare assumes that the needs of individual animals change as they age (Brando and Buchanan-Smith, 2018). In this study, we described age-related variation in hormonal activity (i.e. FAM and FGM concentrations). This suggests that a male’s social and environmental demands will change overtime, requiring that caregivers carefully consider how a male’s age may affect how the animal experiences and responds to physiological and environmental stressors. Beyond population sustainability, there is clearly justification to tailor management practices to individual male elephants to ensure proper animal wellbeing.

In addition to musth episodes, in many of the elephants in this study we also observed periods of elevated FAM concentrations (i.e. elevated FAM episodes) that appeared to be physiologically distinct from musth. These elevated FAM episodes tended to be exhibited by younger elephants compared to confirmed musth episodes, and they were characterized by significantly lower peak FAM concentrations and shorter durations. Although not well-described in the literature, younger male Asian elephants between 8 and 13 years of age regularly exude sweet-smelling odors from the temporal glands that are in stark contrast to the pungent pheromones released by older males during musth (Chandrasekharan *et al.,* 1992; Rasmussen *et al.,* 2002). Termed “moda musth” in the literature (and apparently unique to *E. maximus* compared to *L. africana*), these episodes may serve to physiologically prime younger males while also diffusing potential competitive attention from mature males in musth and/or to offer alternative reproductive strategies for males that would otherwise not gain access to females (Rasmussen *et al.,* 2002). Moda musth has been characterized by intermediate androgen secretion (at higher concentrations than non-musth, but lower concentrations than full musth); some of the elevated FAM episodes described in the present study may represent instances of moda musth. For example, the duration of elevated FAM episodes in this study was inversely related to an elephant’s exposure to male conspecifics. Male elephants undergoing moda musth exhibit avoidance behaviors toward chemical signals from older musth males (Rasmussen *et al.,* 2002), and likewise, social pressures from potential male competitors—especially if they are older—may suppress or shorten periods of elevated androgens. However, we further documented elevated FAM episodes in mature males older than 20 years of age that also exhibited confirmed musth episodes. It is possible that these elevated FAM episodes may be an artifact of various confounding factors that can affect fecal hormone metabolites and the measurement thereof. Androgen concentrations also may be expected to increase in response to heightened sexual activity from conspecifics (Brown *et al.,* 2007; Glaeser *et al.,* 2022). While we do not have detailed information about the sexual state of the female conspecifics of the males in this study (e.g. estrous state or cycling status), it is possible that shorter elevated FAM episodes were responses to reproductive cues from other animals. Mature male Asian elephants in the wild more frequently encounter female or mixed-sex groups during musth (Keerthipriya *et al.,* 2020; LaDue *et al.,* 2022d). Alternatively, elevated FAM episodes may be an artifact of the zoo environment, where elephants may not experience the same socioecological and environmental pressures that would normally suppress excessive androgen secretion and where routinely there is close proximity between males and females that promotes heightened sexual activity (Brown, 2014). Mechanistic and functional explanations of moda musth and other similar elevated sexual states are currently lacking. As more male elephants in *ex-situ* populations reach adolescence, a research opportunity exists to gain a clearer understanding of how and why these periods of heightened androgens exist in wild and/or zoo-housed elephants.

For Asian elephants in human care, there is a pressing need to bolster breeding efforts through innovation in reproductive strategies and techniques to improve the sustainability of *ex-situ* populations (AsERSM, 2017; Fischer, 2017; Bechert *et al.,* 2019). Musth evolved as a male reproductive strategy in elephants to facilitate inter- and intrasexual selection (LaDue *et al.,* 2022b); while it can pose significant husbandry challenges (especially as more males are born in zoos), musth can also be better integrated into animal-centered management plans. To accomplish this, zoo managers need to know how intrinsic and extrinsic factors shape physiological responses around musth. In the present study, we have demonstrated that considerable variation in androgen, glucocorticoid, and thyroid hormone activity exists between and among male Asian elephants housed in zoos, some of which can be attributed to musth. Further, variables such as age, body condition, and social exposure contribute to the physiological responses males exhibit during musth (e.g. hormone concentrations, duration and frequency of musth), suggesting that musth is a flexible occurrence that is sensitive to the interaction between a male’s internal and external environment. Most likely, the inherent variability of musth has ultimate consequences that determine (1) the ability of a male to participate in *ex-situ* breeding efforts (e.g. mate choice, semen quality) and (2) important animal welfare outcomes (e.g. behavioral wellness, physical health). Besides examining the occurrence of similar patterns in zoo-housed African elephants, future studies should focus on investigating other factors that influence musth (e.g. health, nutrition) and the potential behavioral mechanisms that affect physiological responses during musth in *in-situ* and *ex-situ* populations.

## Supplementary Material

Web_Material_coad019

## Data Availability

The data underlying this article will be shared on reasonable request to the corresponding author.
