# Supplementary material for: Hormonal variation and temporal dynamics of musth in Asian elephants (Elephas maximus) are associated with age, body condition and the social environment
Source: Conserv Physiol. 2023 Apr 25;11(1):coad019. doi: 10.1093/conphys/coad019 (PMC10660383; doi:10.1093/conphys/coad019)
Supplement: Web_Material_coad019 [file web_material_coad019.pdf]

Hormonal variation and temporal dynamics of musth in Asian elephants (*Elephas maximus*) are associated with age, body condition, and the social environment

Chase A. LaDue, Kathleen E. Hunt, Wendy K. Kiso, and Elizabeth W. Freeman

Appendix

Table A.1. List of candidate models used during linear mixed model (LMM) approach to identify factors that contribute to variation in FAM, FGM, and FT3 concentrations in male Asian elephants (n = 26). All models included animal and facility identity as random factors.

No.	Model structure
1	$\log_{10}$(Concentration) ~ Null
3	$\log_{10}$(Concentration) ~ Musth status[a]
4	$\log_{10}$(Concentration) ~ Musth status + Age[b]
5	$\log_{10}$(Concentration) ~ Musth status + BCS[c]
6	$\log_{10}$(Concentration) ~ Musth status + (Age × BCS)
7	$\log_{10}$(Concentration) ~ Musth status + (Age × BCS) + (♂ conspecifics[d] × ♂ exposure[e]) + (♀ conspecifics[d] × ♀ exposure[e])

[a] Musth status as measured by FAM concentration: non-musth (reference value), elevated FAM, or musth; not included in FAM model

[b] Age in years, measured at time of sample collection

[d] Body condition score, using standards from Pokharel et al. (2017): BCS 2 (reference value), BCS 3 (reference value, if no BCS 2), BCS 4, or BCS 5

[d] Number of adult male/female conspecifics housed at same facility

[e] Amount of time (to the nearest hour) over the previous 24 h that elephant was in potential tactile proximity to at least one adult male/female conspecific

Table A.2. Ranked regression models investigating effects of various factors on $\log_{10}$-transformed fecal androgen metabolite (FAM), fecal glucocorticoid metabolite (FGM), and fecal triiodothyronine metabolite (FT3) concentrations in male Asian elephants (n = 26). Other statistics include parameterization (k), log-likelihood (LL), Akaike's information criterion score (AIC_c), differences in AIC_c (ΔAIC_c), Akaike weight (w_i), and cumulative Akaike weights (cum. w_i) calculated with restricted maximum likelihood estimation. Refer to Table A.1 for descriptions of each parameters. Asterisk (*) indicate term from the top FAM model that was dropped for non-significance for further analysis; marginal coefficients of determination (R^2_c) are given for the model after non-significant term was dropped. ♂ = male, ♀ = female.

	k	**AIC_c**	**ΔAIC_c**	**w_i**	**LL**	**cum. w_i**
**Fecal androgen metabolites (FAM) (R^2_c = 0.346)**						
(Age × BCS) + (♂ conspecifics* × ♂ exposure) + (♀ conspecifics × ♀ exposure)	17	1190.07	0.00	1.00	−577.79	1.00
(Age × BCS)	11	1205.10	15.03	0.00	−591.44	1.00
BCS	7	1220.74	30.67	0.00	−603.33	1.00
Null model (intercept only)	4	1221.82	31.75	0.00	−606.89	1.00
Age	5	1223.14	33.06	0.00	−606.54	1.00
**Fecal glucocorticoid metabolites (FGM) (R^2_c = 0.299)**						
Musth status + (Age × BCS)	13	−1571.39	0.00	0.52	798.84	0.52
Musth status + Age	7	−1570.65	0.74	0.36	792.37	0.88
Musth status	6	−1567.92	3.47	0.09	789.99	0.97
Musth status + BCS	9	−1565.11	6.28	0.02	791.63	1.00
Musth status + BCS + (♂ conspecifics × ♂ exposure) + (♀ conspecifics × ♀ exposure)	19	−1561.45	9.94	0.00	800.04	1.00
Null model (intercept only)	4	−1537.86	33.52	0.00	772.95	1.00
**Fecal triiodothyronine metabolites (FT3) (R^2_c = 0.439)**						
Null model (intercept only)	4	−321.52	0.00	0.59	164.79	0.59
Musth status	6	−319.09	2.43	0.18	165.62	0.76
Musth status + Age	7	−318.67	2.85	0.14	166.43	0.91
Musth status + (Age × BCS)	19	−317.38	4.14	0.07	178.37	0.98
Musth status + BCS + (♂ conspecifics × ♂ exposure) + (♀ conspecifics × ♀ exposure)	9	−314.16	7.36	0.01	166.24	0.99
Musth status + (Age × BCS)	13	−312.11	9.41	0.01	169.38	1.00
